# Design of MAPb(Br_x_I_1−x_)_3_-Based Solar Cells: Compositional Optimization for Thermally Stable and Defect-Tolerant Devices

**DOI:** 10.3390/nano16150965

**Published:** 2026-08-06

**Authors:** Syed Abdul Moiz, Muhammad I. Masud, Muhammad Kashif

**Affiliations:** 1 Device Simulation Laboratory, Department of Electrical Engineering, College of Engineering and Architecture, Umm Al-Qura University, Makkah 21955, Saudi Arabia; 2 Department of Electrical Engineering, College of Engineering, University of Business and Technology, Jeddah 21361, Saudi Arabia; m.masud@ubt.edu.sa; 3 School of Electrical and Information Engineering, Tianjin University, 92 Weijin Road, Nankai District, Tianjin 300072, China; mkashif@tju.edu.cn

**Keywords:** solar cell, perovskite, MAPb(Br_x_I_1−x_)_3_, PEDOT:PSS, TiO_2_, SCAPS-1D

## Abstract

Mixed halide perovskites can be tuned for bandgap, but they are prone to thermal defect deterioration that is difficult to evaluate throughout the whole stoichiometry range. Here, we investigate the relationship between Br composition, trap density, and temperature. It is observed that asymmetric thermal defect trade-offs provide predictive design principles beyond obvious efficiency trends. We use SCAPS-1D to model MAPb(Br_x_I_1−x_)_3_ solar cells. Simulation parameters include continuous bowing-corrected functions of Br fraction (0 ≤ x ≤ 1), temperature (300–350 K), and trap density (10^11^–10^20^ cm^−3^). Despite a trade-off between short circuit current and open circuit voltage with Br incorporation (power conversion efficiency drops from ~25.5% at x = 0 to ~12% at x = 1), important results reveal non-trivial asymmetries: (i) Br-rich compositions are more sensitive to trap-assisted SRH recombination at Nt > 10^18^ cm^−3^ than I-rich absorbers; (ii) the thermal degradation coefficient dVoc/dT is lower for Br-rich systems than for I-rich systems, indicating improved thermal tolerance for Br-rich systems; and (iii) these quantitative design guidelines give predicted assistance for producing mixed halide perovskite devices with higher operational stability.

## 1. Introduction

According to the recent NREL efficiency chart, lead-based perovskite solar cells have achieved more than 28% power conversion efficiency in single-junction configurations, thanks to extensive research efforts all around the world [1,2]. Now researchers are optimistic about the perovskite solar cell industry’s future expansion [3,4]. But many researchers believe that lead-based toxicity will be the principal barrier to commercializing perovskite solar cells. However, lead-based toxicity can be minimized by using chemical additives, waterproof or self-healing encapsulation, partial lead replacement with suitable metals, halide mixing with bromide, or non-toxic metal substitution [5,6,7,8,9,10,11,12,13,14,15].

Compositional engineering of perovskite involves substituting lead ions in the crystalline structure of ABX_3_ to minimize toxicity. Methylammonium lead iodide (MAPbI_3_) is a very effective and well-researched perovskite material for compositional engineering [16]. However, the restricted tunability of the bandgap in MAPbI_3_ limits the possibilities for improving optical properties compared to halide mixing. Generally, the perovskite absorber MAPbI_3_ is sensitive to degradation and phase transition at high temperature, as well as after water molecule adsorption [17]. This inherent instability is a significant challenge for long-term device operation and needs to be tackled through stable perovskite compositions or encapsulation strategies for practical viability.

The energy bandgap of MAPb(Br_x_I_1−x_)_3_ may be controlled from 1.55 eV to around 2.3 eV by altering the bromide content (x), making it promising for efficient solar cells. Compositional engineering of MAPb(Br_x_I_1−x_)_3_ alters the structural, electrical, mechanical strain, optical, and thermal characteristics as the Br level increases. The optimal Br concentration for overcoming the inherent instability of MAPb(Br_x_I_1−x_)_3_ through compositional engineering is a highly discussed issue in recent literature [18,19,20].

Many previous numerical studies neglected to account for the important interaction that occurs when these parameters, such as compositional tuning, thermal effects, and defect density, are changed simultaneously. The purpose of this study is to address this gap by modeling the compositional tuning, thermal effects, and defect density (x, T, and Nt) dependence of Voc, Jsc, FF, and PCE. It then parameterizes all significant photovoltaic properties as continuous, bowing-corrected functions of Br fraction x across the whole compositional range (0 ≤ x ≤ 1). This work investigates the temperature-dependent electrical performance of MAPb(Br_x_I_1−x_)_3_-based devices. These insights may help create compositions with lower thermal sensitivity. However, thorough stability evaluation requires further experimental confirmation of the real degradation processes [20,21,22,23,24,25].

The toxicity and stability of MAPb(Br_x_I_1−x_)_3_ perovskite may be influenced by the relative concentration of Br and I because of structural and chemical changes in mixed halide systems. Numerous experimental studies have shown that the incorporation of Br can improve the stability of the material with respect to moisture and phase transitions [17,18,19], but the overall toxicity is less clear (partial replacement of Br reduces the iodine content, but lead is the main toxic species). This numerical study complements this understanding by quantifying the role of Br concentration (x) in tuning the defect tolerance and thermal resilience and helps to identify compositions that can not only balance efficiency and stability, but also reduce the dependence on toxic lead through compositional optimization.

In this study, a comprehensive numerical analysis of absorber MAPb(Br_x_I_1−x_)_3_-based perovskite solar cells is carried out for the whole absorber compositional (0 ≤ x ≤ 1) range, with TiO_2_ as the electron transport layer (ETL) and PEDOT:PSS as the hole transport layer (HTL). The parameters for all important optoelectronic material characteristics, including bandgap, electron affinity, carrier mobility, and dielectric permittivity, are defined as continuous functions of Br percent x that account for bending correction, according to previous experimental evidence. To fully evaluate the contributions of recombination across all compositions, the operating temperature is systematically changed from 300 to 350 K, and the trap density is swept from 10^11^ to 10^20^ cm^−3^. The following variables are taken into consideration and examined as interdependent functions of halide composition x, temperature T, and trap density N_t_: open circuit voltage (V_oc_), short circuit current density (Jsc), fill factor (FF), and power conversion efficiency (PCE). In order to create thermally stable and defect-tolerant MAPb(Br_x_I_1−x_)_3_ perovskite photovoltaic devices that can withstand real-world operating conditions, the results that were obtained reveal composition-dependent asymmetries in thermal resilience and sensitivity between Br-rich and I-rich compositions.

The energy bandgap and electron affinity of perovskite materials are critically determined by the ratio of Br and I concentration. Substitution of iodide by bromide leads to an increased bandgap by a downshift of the valence band maximum. This is mainly caused by halide p-orbital contributions [26]. Such tunability has a direct impact on the ionization potential and electron affinity that characterize the absolute energy levels. The device performance is strongly dependent on the optimal band alignment at the interfaces of the perovskite/charge transport layers. Conduction band offsets of 0.0–0.3 eV and appropriately positioned valence bands minimize recombination and permit efficient charge collection. This is borne out by theoretical analysis [27]. This results in a “cascade” band alignment that facilitates smooth charge transport, as opposed to detrimental “cliff” or “spike” alignments [28]. So, halide composition engineering is a powerful tool for both tuning the bandgap and optimizing the energy landscape of the whole device.

To the best of authors’ knowledge, the combined interdependencies of bromide composition, trap density, and operating temperature on the photovoltaic performance of MAPb(Br_x_I_1−x_)_3_ perovskite solar cells have not been investigated before. While a number of SCAPS-1D-based studies have been performed to explore compositional engineering, defect density, and temperature effects in perovskite systems [20,21,22,23,24,25], these factors have often been examined in isolation or over a limited compositional range. For example, mixed halide MAPb(I_1−x_Cl_x_)_3_ devices were improved by Paz Totolhua et al. by tuning thickness, defect density, and contact work function [29]. Some studies have been conducted on MAPbI_3_ performance as a function of temperature or effects of chloride inclusion [30,31]. However, none of these studies consider the interdependence of full-range halide composition (x = 0 to 1), wide-range trap density (10^11^–10^20^ cm^−3^), and operating temperature (300–350 K) in a single framework. This work aims to fill the gap by simulating the compositional tuning, thermal effects, and defect density dependency of open circuit voltage, short circuit current, fill factor, and power conversion efficiency throughout the entire compositional range. All the key optoelectronic material parameters, such as the bandgap, electron affinity, carrier mobility, and dielectric permittivity, are parameterized as continuous bowing-corrected functions of Br percent x, based on experimental data. The comprehensive coupled study exposes the composition-dependent asymmetry of heat resilience and trap sensitivity between Br-rich and I-rich compositions, which was absent in single-variable experiments. This work offers insights into the temperature dependent electrical performance of MAPb(Br_x_I_1−x_)_3_-based devices and may guide the development of lower thermally sensitive compositions. However, detailed stability assessment requires additional experimental confirmation of degradation pathways.

## 2. Simulation Methods and Device Structure

### 2.1. SCAPS-1D Simulation Framework

SCAPS-1D was selected for this study because SCAPS-1D (i) is a physics-based simulator for multilayer thin-film devices; (ii) solves carrier transport and recombination (e.g., SRH, radiative, and Auger); (iii) supports defect and interface modeling; (iv) predicts key performance metrics used in studies; (v) provides carrier/recombination profiles and band diagrams; (vi) enables sensitivity studies and identification of dominant loss mechanisms; (vii) supports realistic stack representation (thickness, doping, and mobilities) with boundary/contact conditions; (viii) allows calibration to experimental data; and (ix) enables fast, cost-effective optimization and literature-comparable modeling assumptions [32,33,34,35].

### 2.2. Mathematical Models Employed for Simulation

The complete mathematical formulations of the semiconductor transport and recombination models employed in this study, along with the full list of investigated device compositions, are provided in the Supplementary Materials (Equations (S1)–(S15) and Table S1) [36,37,38,39,40,41,42,43,44,45,46,47,48].

**Figure 1:**
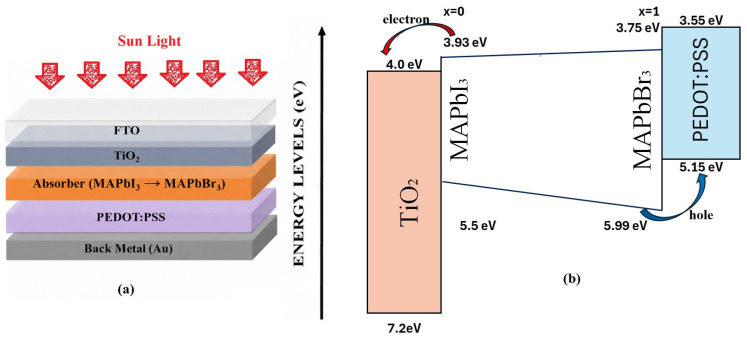
(**a**) Cross-section architecture of the perovskite solar cell (FTO/TiO_2_/MAPb(I_1−x_Br_x_)_3_/PEDOT:PSS/Au). (**b**) Corresponding energy level diagram showing electron and hole transport pathways.

To facilitate a clear comparison, the electron transport layer and hole transport layer are standardized across all eleven devices, using titanium dioxide (TiO_2_) for the electron transport layer and poly(3,4-ethylenedioxythiophene):poly(styrene sulfonate) PEDOT:PSS) for the hole transport layer. All eleven devices use the standard n-i-p planar heterojunction architecture: FTO/TiO_2_/MAPb(Br_x_I_1−x_)_3_/PEDOT:PSS/Au, as shown in Figure 1. The essential optoelectronic material characteristics such as bandgap, electron affinity, carrier mobility, and dielectric permittivity are expressed as continuous, bowing-corrected functions of the Br fraction x, grounded in proven experimental data. The operating temperature ranges from 300 K to 350 K, and the trap density varies from 10^11^ to 10^20^ cm^3^ to comprehensively assess the contributions of Shockley–Read–Hall recombination across all compositions [49,50].

### 2.3. Simulation Material Parameters

All material properties used in the SCAPS-1D simulations are derived from well-accepted experimental and theoretical literature to guarantee physical correctness and repeatability [49,50,51,52,53,54,55,56,57,58,59,60,61,62,63,64]. The data for the fixed transport layers, namely the TiO_2_ electron transport layer (ETL) and PEDOT:PSS hole transport layer (HTL), including bandgap, electron affinity, carrier mobility, effective density of states, and doping concentrations, are sourced directly from extensively referenced SCAPS-1D perovskite simulation studies. For the MAPb(Br_x_I_1−x_)_3_ absorber layer, all essential optoelectronic characteristics are characterized as linear, composition-dependent functions of the bromide fraction x through Vegard’s law with an optical bowing correction, which is commonly described as Equation (1) [55,56].

(1) $$P\left(x\right)=P_{I}\;\left(1-x\right)+P_{Br}\left(x\right)-b_{p}(1-x)$$

Here, P(x) denotes a composition-dependent material property, with P_I_ and P_Br_ representing the values of the pure iodide (MAPbI_3_) and pure bromide (MAPbBr_3_) end members, respectively. The parameter b_P_ is specific to each property. The bandgap and electron affinity are calculated using experimentally validated bowing parameters of b_Eg_ = 0.33 eV and b_χ_ = 0.06 V, respectively, while the relative dielectric permittivity εr and carrier mobilities μ_n_ and μ_p_ are linearly interpolated between the end member values. For optical modeling, the SCAPS-1D software has several algorithms that may support multiple designs for forecasting the absorption coefficient [49,56,60,61,62,63,64].

Similarly, the given Equation (2) is a commonly used computational approach for evaluating the optical absorption coefficient model for any type of solar cell, including perovskite solar cells (PSCs), where A and B are constants, h is a Planck constant, ν represents the photon frequency, and Eg is the energy bandgap of the absorber material [40]:

(2) $$\alpha =A+\frac{A}{h\vartheta }\sqrt{h\vartheta -E_{g}}$$

Table 1 and Table 2 summarize all the input-required physical and material parameters that were used in the proposed simulation study.

### 2.4. Simulation Framework

Firstly, both transport layers, TiO_2_ and PEDOT:PSS, are optimized in terms of thickness and doping density as a precondition for research, and then the MAPb(Br_x_I_1−x_)_3_ layer thickness is varied from 100 nm to 1000 nm in uniform increments to identify the optimal thickness that maximizes power conversion efficiency for each device. The bulk trap density N_t_ is independently swept from 10^11^ cm^−3^ to 10^20^ cm^−3^ at mid-gap energy to quantify Shockley–Read–Hall recombination contributions as a function of halide composition x.

**Figure 2:**
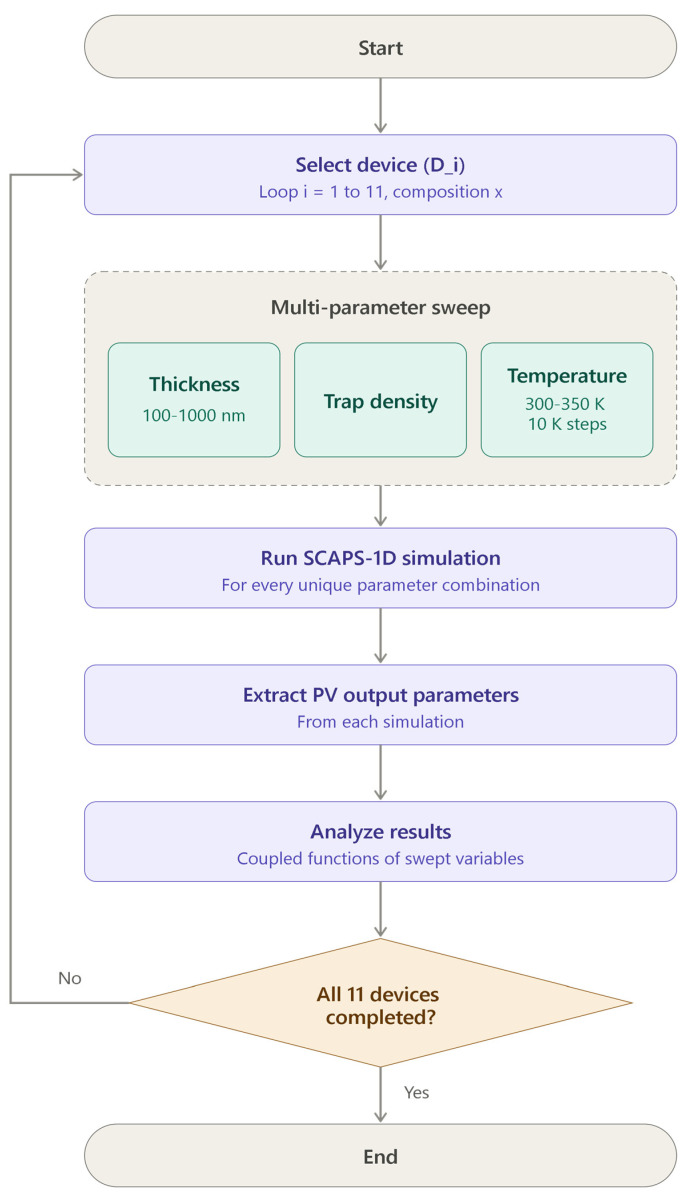
Systematic simulation flowchart for the proposed photovoltaic devices based on perovskite absorber MAPb(Br_x_I_1−x_)_3_ for D1 to D11 devices.

Operating temperature is systematically varied from 300 K to 350 K in steps of 10 K to evaluate thermally activated carrier transport, ion migration, and recombination rate enhancement across all compositions. Additional recombination parameters investigated include the carrier lifetimes and diffusion coefficient. The extracted photovoltaic output parameters, open circuit voltage, short circuit current, fill factor, and power conversion efficiency are analyzed as coupled functions of all swept variables to construct a complete performance landscape for each of the eleven absorber compositions. The step-by-step simulation procedure used in this study is illustrated in Figure 2.

The device architecture used in this work is FTO/TiO_2_/Perovskite/PEDOT:PSS/Au, which is a regular n-i-p architecture with TiO_2_ as the electron transport layer and PEDOT:PSS as the hole transport layer. PEDOT:PSS is mostly used in inverted (p-i-n) configurations, but its application in normal n-i-p architectures has been experimentally demonstrated by different processing methods such as water-free PEDOT:PSS dispersions [72], WO_3_/PEDOT:PSS composites (15.1% efficiency) [73], solvent-free transfer lamination [74], ALD-assisted interfacial engineering (11% efficiency) [75], toluene-dispersed PEDOT ink (14.5% efficiency) [76], alcohol-dispersed PEDOT:F complexes (24.5% efficiency) [77], transfer printing [78], and dopant-free spiro-OMeTAD/PEDOT mixtures (18.7% efficiency) [79].

The key requirement for any hole transport layer in an n-i-p configuration is energy level alignment with the perovskite valence band, and photoelectron spectroscopy has confirmed the type II band alignment of the heterojunction, enabling efficient hole extraction and electron blocking [49,50,51,52,53]. Importantly, our central objective—investigating the coupled effects of bromide composition, trap density, and temperature on recombination dynamics and performance—is architecture-independent, as the physical principles governing defect tolerance, thermal resilience, and composition-dependent asymmetries are fundamental material properties applicable to both n-i-p and p-i-n configurations. All simulation parameters are derived from experimentally validated material properties.

## 3. Results

### 3.1. Photocurrent–Voltage Responses of the Proposed Devices

**Figure 3:**
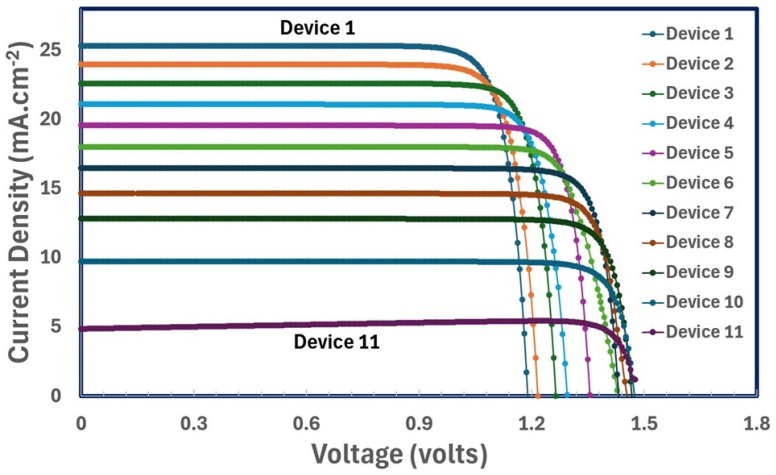
Current density–voltage (J–V) characteristics of MAPb(Br_x_I_1−x_)_3_-based perovskite solar cells for devices D1–D11 with varying bromide fraction (x = 0.0–1.0).

Photocurrent–voltage characterization is one of the most important techniques for analyzing the overall behavior of the basic photovoltaic solar cell. Figure 3 depicts the overall photovoltaic response as current–voltage (J–V) characteristics of MAPb(Br_x_I_1−x_)_3_ perovskite solar cells throughout the whole compositional range, from x = 0 to 1. All devices exhibit the typical photovoltaic behavior of solar cells. Where a smooth photocurrent plateau is formed at low forward bias, it is followed by a sharp decline approaching the open circuit voltage, signifying efficient carrier collection under short circuit conditions and diode-dominated recombination near the open circuit voltage. A consistent trade-off is observable in the compositions, as the increasing bromide content (x) progressively reduces the short circuit current density, consistent with the widening optical bandgap that blue-shifts the absorption onset and narrows the spectral collection range. Simultaneously, the J–V curve advances to higher voltages as x increases, indicating an enhanced open circuit voltage due to the increased quasi-Fermi level splitting in wider-gap absorbers. The interplay between the declining photocurrent and the rising voltage output dictates the overall power conversion efficiency of any composition [80,81].

### 3.2. Effects of Bromide Concentration on Photovoltaic Performance Parameters

**Figure 4:**
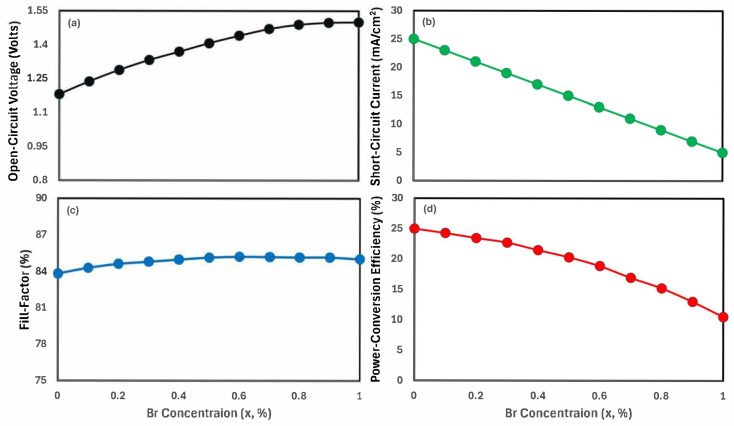
Photovoltaic performance parameters of MAPb(Br_x_I_1−x_)_3_-based devices (D1–D11) as a function of bromide fraction (x). (**a**) Open circuit voltage, (**b**) short circuit current density, (**c**) fill factor, and (**d**) power conversion efficiency.

Figure 4 illustrates the photovoltaic performance parameters, such as (a) open circuit voltage, (b) short circuit current, (c) fill factor, and (d) power conversion efficiency of MAPb(Br_x_I_1−x_)_3_-based devices (D1–D11). The figure clearly demonstrates that all devices exhibit a clear dependence on the bromide fraction x, which increases from 0 to 1.0. As the Br content (x) of MAPb(Br_x_I_1−x_)_3_ increases, the open circuit voltage (see Figure 4a) shows a steady enhancement from ~1.18 V (D1, x = 0.0) to ~1.5 V (D11, x = 1.0). This improvement is attributed, maybe, to the widening of the perovskite bandgap with increasing bromide incorporation [59]. Conversely, the short circuit current density (see Figure 4b) decreases monotonically from ~24.5 mA·cm^−2^ to ~9.5 mA·cm^−2^ over the same compositional range as x, reflecting reduced photon absorption due to the broader bandgap. The fill factor (see Figure 4c) also shows a small but steady improvement, going from about ~83.8% at x = 0 to about ~86.9% at almost x = 0.7 and then going down to about ~86.3% at x = 1. This means that more charge is being extracted and less charge is being lost through recombination at higher bromide fractions. However, the power conversion efficiency shows a decreasing trend (see Figure 4d). The power conversion efficiency declines from ~25.5% at x = 0.0 (MAPbI_3_) to ~12% at x = 1 (MAPbBr_3_), mainly due to the significant reduction in photocurrent despite the gains in open circuit voltage and fill factor, as shown in Figure 4a,c. These results clearly demonstrate that intermediate compositions of the Br fraction (x) cause a decrease in the power conversion efficiency performance of photovoltaic devices [60].

### 3.3. Effects of Bromide Concentration on External Quantum Efficiency

**Figure 5:**
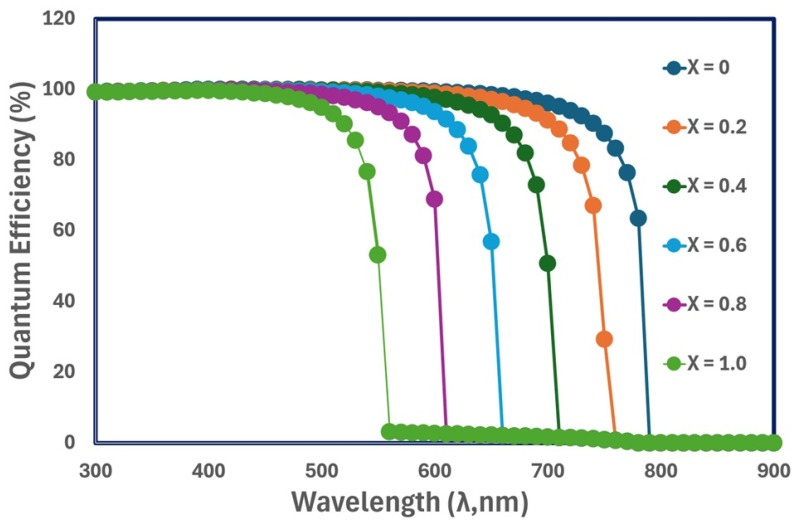
External quantum efficiency (EQE) spectra of the selected MAPb(Br_x_I_1−x_)_3_ devices (x = 0.0, 0.2, 0.4, 0.6, 0.8, and 1.0) as a function of wavelength.

Photocurrent–voltage characterization does not convey the complete performance picture of the proposed photovoltaic devices. Therefore, external quantum efficiency analysis is performed for correlating the compositionally dependent optical absorption with the electrical output, as present in Figure 5. The external quantum efficiency spectra for selected devices (x = 0.0 (MAPbI_3_), 0.2, 0.4, 0.6, 0.8, and 1.0 (MAPbBr_3_)) exhibit a gradual blue shift in the absorption onset with increasing bromide content, which is consistent with the widening of the bandgap [82]. It is further observed that all devices effectively collect free charge carriers at their respective electrodes with negligible recombination losses, since the external quantum efficiency is more than 80% throughout a broad range of wavelengths [83]. The intermediate composition range between x = 0 and x = 1 has a slightly better external quantum efficiency response in comparison to longer wavelengths. Such improvement might be due to better film quality or decreased fault density. Overall, the integrated EQE spectra yield short circuit current values that are in excellent agreement with those derived from the photocurrent–voltage characteristics, thereby confirming the reliability of the measured photocurrents across all devices.

### 3.4. Effects of Trap Density on Photovoltaic Performance Parameters

**Figure 6:**
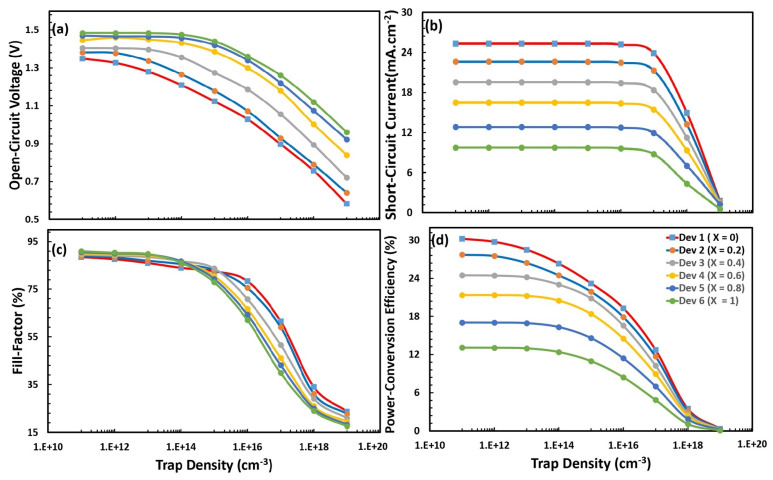
Photovoltaic parameters. (**a**) Open circuit voltage, (**b**) short circuit current, (**c**) fill factor, and (**d**) power conversion efficiency of MAPb(Br_x_I_1−x_)_3_ devices (x = 0.0, 0.2, 0.4, 0.6, 0.8, and 1.0) as a function of bulk trap density ($N_{t}$).

Trap density analysis is crucial for measuring photovoltaic devices performance, because it helps understand the defects’ behavior that causes the electron–hole recombination process. The efficient control of the recombination process in terms of defect density plays a vital role in minimizing energy loss and maximizing the cell’s power conversion efficiency [84,85]. Therefore, in order to investigate bulk defects affecting device performance, the trap density (N_t_) in the absorber layer MAPb(Br_x_I_1−x_)_3_ was systematically changed for certain compositions of Br (x = 0.0, 0.2, 0.4, 0.6, 0.8, and 1.0) from 10^11^ to 10^20^ cm^−3^, as illustrated in Figure 6a–d. The figures clearly established that all compositions exhibit very similar trends for (a) open circuit voltage, (b) short circuit current density, (c) fill factor (FF), and (d) power conversion efficiency across the whole trap density range. At the highest defect density (N_t_ > 10^18^ cm^−3^), photovoltaic parameters, especially power conversion efficiency, show slight variations for each device.

**Figure 7:**
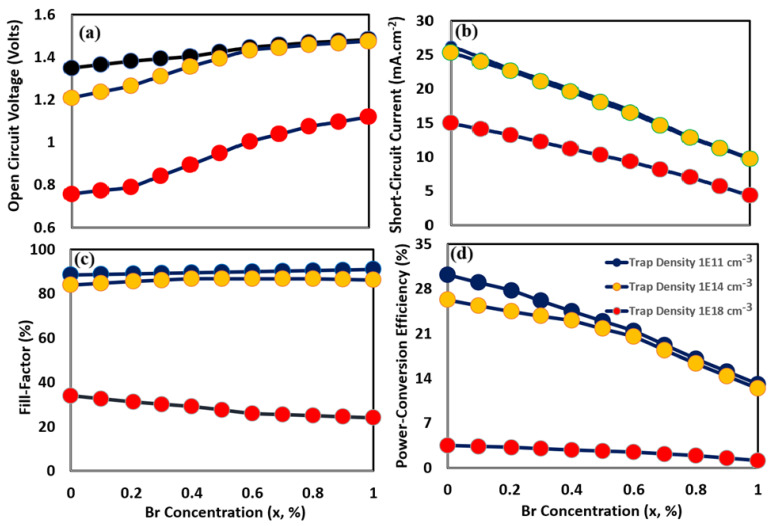
Photovoltaic parameters. (**a**) Open circuit voltage, (**b**) short circuit current, (**c**) fill factor, and (**d**) power conversion efficiency of MAPb(Br_x_I_1−x_)_3_ devices as a function of Br concentration (x) at three levels of bulk trap density ($N_{t}$): 10^11^, 10^14^, and 10^18^ cm^−3^.

To further elaborate on the impact of Br contents (x) on photovoltaic device performance, the photovoltaic parameters were simulated across a range of Br contents (x) at three defect levels in Figure 7. As shown in the figure, the (a) open circuit voltage, (b) short circuit current density, and (c) fill factor remain largely stable at low-to-moderate trap densities (from 10^11^ up to 10^14^ cm^−3^), which indicates that the devices exhibit reasonable tolerance to bulk recombination centers [86]. However, at higher trap densities (10^18^−10^19^ cm^−3^), a pronounced degradation in all performance metrics is observed, which may be attributed to enhanced Shockley–Read–Hall recombination that reduces the carrier extraction efficiency [87]. The decay amount seems to change with the bromide level, which may mean that the Br composition affects defect tolerance. High trap density generates a large density of electronic energy levels inside the forbidden bandgap. Photogenerated electrons and holes quickly fill these trap states instead of remaining free in the conduction and valence bands. This immobilizes the charges and “pins” the quasi-Fermi levels to the trap energy levels [88], which in turn causes the devices to exhibit almost identical power conversion efficiency (PCE) as a function of Br concentration (x), as observed in Figure 7.

### 3.5. Effects of Trap Density on Saturation Current

**Figure 8:**
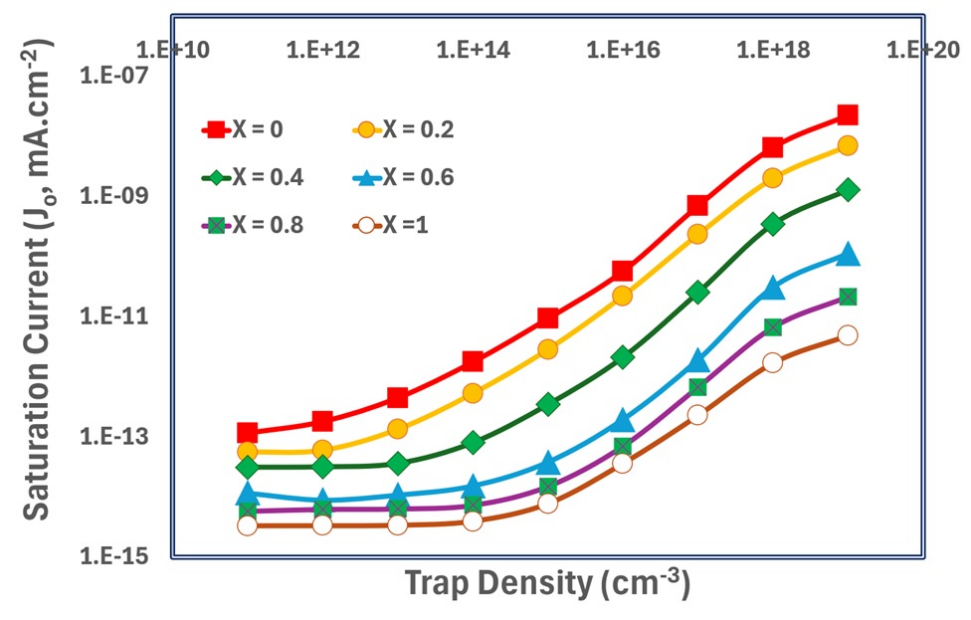
Saturation current density (J_o_) of the selected MAPb(Br_x_I_1−x_)_3_-based perovskite photovoltaic devices (x = 0.0, 0.2, 0.4, 0.6, 0.8, and 1.0) as a function of trap density.

Saturation current density (Jo) is another important parameter used to measure the performance of perovskite solar cells. Saturation current density controls the open circuit voltage and qualitatively reflects the internal recombination losses. Therefore, reducing the saturation current density as much as possible is the main objective for efficient photovoltaic design. The mathematical relation between open circuit voltage and saturation current density can be defined as Equation (3) [37,38]:

(3) $$V_{OC}=\frac{nkT}{q}\;ln\;\left(\frac{J_{SC}}{J_{o}}+1\right)$$

where n is the diode ideality factor. The diode ideality factor indicates the extent to which the solar cell’s current–voltage characteristics deviate from the ideal diode’s equation. In most cases, the diode ideality factor is very close to 1.5 for many perovskite solar cells [89]. As in Figure 6, we have already determined the short circuit current and open circuit voltage for trap densities from 10^11^ to 10^20^. Therefore, J_0_ is calculated as a function of trap density from Equation (4), and the output response is displayed in Figure 8 [37,38].

(4) $$J_{O}=\frac{J_{sc}}{exp\left(\frac{qV_{oc}}{nkT}\right)-1}$$

The impact of bulk defects in the absorber layer, MAPb(Br_x_I_1−x_)_3_, on charge carrier dynamics was investigated by plotting the saturation current density (J_0_) against trap density (N_t_) over the compositional concentration of Br in terms of x. All bromide fractions show a monotonic rise in J_0_ with increased Nt, as shown in Figure 8. This agrees with the estimated increment in dark current mediated by Shockley–Read–Hall recombination [90]. At all trap densities, the saturation current is rather constant regardless of the halide content, indicating that the recombination kinetics are controlled more by the concentration of defects than by mobility or bandgap fluctuations in mixed halide systems [91,92], as observed for many other devices.

### 3.6. Effects of Br Concentration on Carrier Lifetime

Free electron and hole pairs are generated inside the perovskite absorber layer in response to light striking a perovskite solar cell. These pairs have a high probability of recombination after traveling a certain distance inside the absorption layer. Therefore, carrier lifetime is defined as the average amount of time an electron and a hole remain as free carriers prior to recombination inside the absorber layer [93]. Free electron and hole pairs are generated inside the perovskite absorber layer in response to light striking a perovskite solar cell. These pairs have a high probability of recombination after traveling a certain distance. Longer carrier lifetimes are associated with longer diffusion lengths, which in turn reduce recombination losses and increase open circuit voltage and short circuit current density, respectively, making perovskite solar cells more efficient.

The carrier lifetime (τ) and saturation current density (J_0_) are correlated as [94,95]:

(5) $$J_{O}=\frac{qn_{i}}{2}\sqrt{\frac{D}{\tau }}$$

**Figure 9:**
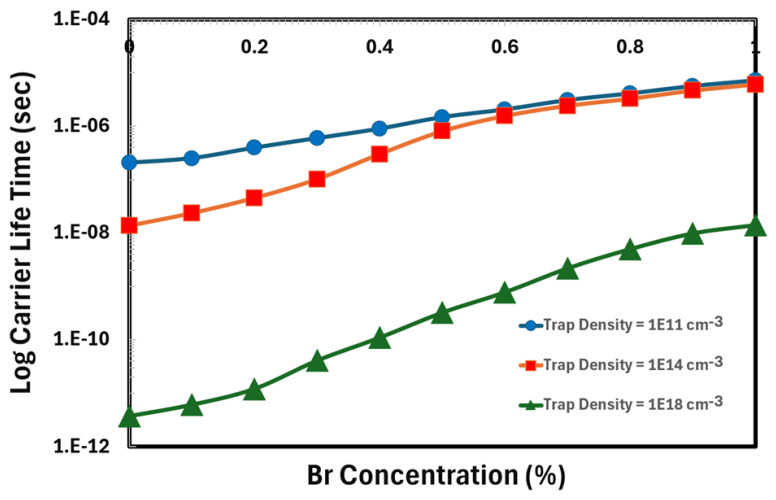
Carrier lifetime (τ) of MAPb(Br_x_I_1−x_)_3_-based perovskite photovoltaic devices at trap densities of 10^11^, 10^12^, and 10^18^ cm^−3^ (as a function of trap density).

As we already know from Figure 8, the saturation current density is a function of Br concentration (x) for MAPb(Br_x_I_1−x_)_3_. Therefore, the carrier lifetime is also a function of Br concentration (x) for MAPb(Br_x_I_1−x_)_3_, which is calculated from the above Equation (5), and the output response is present in Figure 9 at various trap densities.

The figure shows that the lifetime of carriers is related to how much bromide is in the absorber layer and the number of defects present. Throughout the whole range of Br concentrations, the carrier lifetime for devices with a relatively lower trap density of 10^11^ cm^−3^ is continuously high, on the order of ~10^−7^ s at x = 0 (MAPbI_3_), and more or less linearly increases up to ~10^−6^ s for x = 1 (MAPbBr_3_). Even at a trap density of 10^14^ cm^−3^, the carrier lifetime is approximately 10^−8^ s at x = 0 (MAPbI_3_) and increases quasi-linearly to about 10^−6^ s at x = 1 (MAPbBr_3_). Both trends are very close after a Br concentration of 0.5, which shows that the recombination dynamics in high-quality films are mostly determined by the intrinsic material properties, rather than the Br concentration in the absorber layer MAPb(Br_x_I_1−x_)_3_. With an increase in trap density to 10^14^ cm^−3^ inside the absorber layer, there is a slight decrease in carrier lifetime for certain compositions, suggesting that defect-assisted Shockley–Read–Hall recombination starts to be more important. At the maximum defect density of 10^18^ cm^−3^, the carrier lifetime declines to 10^−12^ to 10^−9^ s, showing the essential dominance of defect-assisted Shockley–Read–Hall recombination. The results suggest that the energy spectrum of the defects may be affected by bromide inclusion, but the existence of a dense and deep level of traps seems to completely nullify its mitigating impact [94,95,96].

### 3.7. Effects of Br Concentration on Carrier Diffusion Length (L) and Diffusion Coefficient (D)

The diffusion coefficient (D) is another important parameter used to measure the ease with which free charge carriers can move through a layer with a concentration gradient. Similarly, the carrier diffusion length (L) is the average distance a free carrier travels before the recombination process. The carrier lifetime (τ) is the fundamental link between these two parameters [38]:

(6) $$L=\sqrt{D\tau }$$

where D is calculated through Einstein’s relation from Equation (S7).

**Figure 10:**
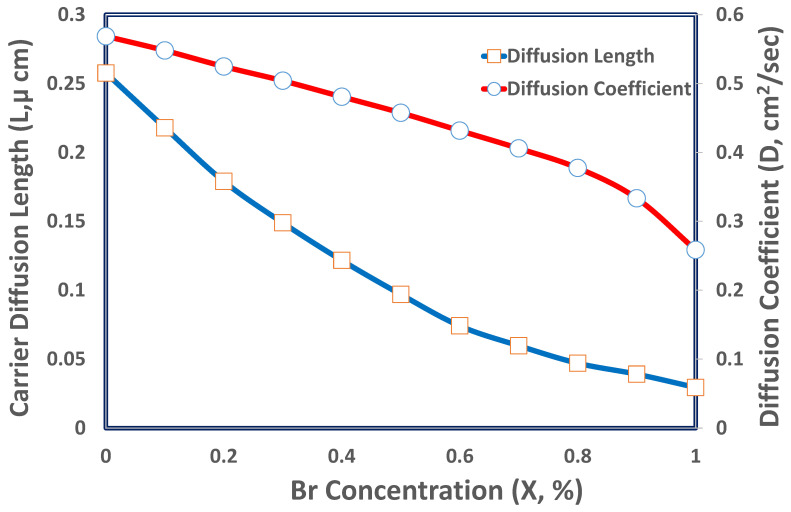
Carrier diffusion length (L) and diffusion coefficient (D) of MAPb(Br_x_I_1−x_)_3_-based perovskite photovoltaic devices as a function of Br concentration.

Figure 10 shows that both carrier diffusion length and diffusion coefficient have very similar trends, monotonically decreasing with increasing bromide concentration. At a Br concentration of x = 0 (MAPbI_3_), the diffusion length is approximately 0.26 μm with a corresponding diffusion coefficient of 0.57 cm^2^/s; however, as the Br fraction increases, both parameters steadily decline, reaching a diffusion length of ~0.02 and a diffusion coefficient of 0.26 cm^2^/s at Br concentration x = 1 (MAPbBr_3_). The trends might be explained by the fact that the inclusion of bromide causes more free carrier scattering and structural disorder, which in turn increases carrier trapping and decreases mobility. The above relation (Equation (6)) suggests that the degradation in carrier diffusion length is driven mainly by a reduction in diffusion coefficient, rather than a relatively minor change in lifetime. The lowest value of both diffusion coefficient and carrier diffusion length at higher Br concentrations (x = 1) indicates that the transport properties are compromised. This may be due to phase segregation or the formation of deep trap states in the presence of a higher concentration of Br in the absorber layer [97].

The aim of including halide in perovskite solar cells is usually to widen the bandgap, and we looked at how this affects open circuit voltage and short circuit current. Through rigorous device simulations, we demonstrate that the synergistic effect of the Br/I ratio on both bandgap and electron affinity may generate non-linear interfacial band offsets, resulting in unforeseen recombination pathways. We observe that interfacial barriers and bulk properties often limit performance when using a full balancing framework. We identify device-specific anomalies, such as unexplained reductions in fill factor that cannot be explained by bandgap scaling, and attribute these to field-dependent charge extraction and trap-assisted recombination at selected interfaces. We distinguish between parameter-driven expectations and actual physical results, thus gaining a better understanding of the halide-dependent device physics. Our work validates Br/I engineering as a tactical strategy to optimize the overall energy framework, providing design principles for high-efficiency perovskite solar cells.

### 3.8. Effects of Temperature on the Photovoltaic Responses of the Devices

**Figure 11:**
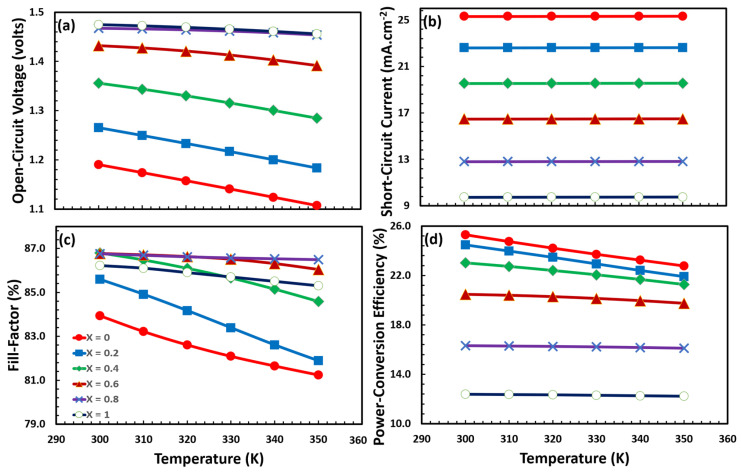
Temperature-dependent photovoltaic performance of MAPb(Br_x_I_1−x_)_3_-based perovskite solar cells: open circuit voltage (**a**), short circuit current density (**b**), fill factor (**c**), and power conversion efficiency (**d**) as a function of operating temperature from 300 K to 350 K.

The photovoltaic parameter behavior of MAPb(Br_x_I_1−x_)_3_-based devices as a function of temperature is shown in Figure 11. The figure demonstrates clear temperature-dependent degradation across the measured range of 300 K to 350 K for all devices, especially at lower values of x (Br concentration), with a particular emphasis on the open circuit voltage and fill factor of the devices. As the temperature rises, the open circuit voltage (see Figure 11a) drops gradually in a very similar trend independent of Br concentration (x). This degradation of open circuit voltage with increasing temperature arises from the exponential increase in the reverse saturation current density and causes Shockley–Read–Hall recombination to enhance [98]. In contrast, the short circuit current (see Figure 11b) remains stable throughout the given temperature range (300 to 350 °C) for all concentrations (x) of Br, because at this temperature range the primary factors governing the perovskite solar cells are photocurrent generation, the absorption coefficient, and the energy bandgap. All of these factors exhibit only minor variations (nearly negligible) at this temperature range, suggesting that carrier generation and collection efficiency are largely insensitive to variations within this temperature range. The fill factor shows a slight decline (Figure 11c), while the power conversion efficiency (Figure 11d) decreases correspondingly from 3.1% to 2.5%, reflecting the combined effect of reduced voltage and fill factor at elevated temperatures. These observations may be attributed to enhanced phonon scattering and increased trap-assisted recombination at higher temperatures, which collectively degrade device performance [98,99,100].

## 4. Conclusions

While MAPb(Br_x_I_1−x_)_3_’s compositional flexibility provides a way forward for bandgap engineering, the interplay among halide stoichiometry, thermal stress, carrier dynamics, and defect density is still mostly unknown. By conducting rigorous simulations over the entire Br fraction range, this study systematically maps these interdependencies and uncovers a basic trade-off: as Br enrichment increases, it is important to consider that the bandgap widens. This, in turn, increases open circuit voltage to around 1.5 V while simultaneously decreasing short circuit current from about ~24.5 to about ~9.5 mA·cm^−2^ and finally lowering power conversion efficiency from about ~25.5% to about ~12%. Crucially, compositions rich in Br had less temperature-induced degradation of open circuit voltage and fill factor in the 300–350 K range, while absorbers rich in I had stronger photocurrent but greater thermal sensitivity of the fill factor. However, these results are confined to steady-state electrical simulations and do not account for long-term degradation processes, including ion migration, phase segregation, or halide volatilization. As a result, the observed trends should be understood as temperature-dependent performance differences rather than overall thermal stability.

Similarly, carrier transport is decreased in bromide-dominated systems, which is counteracted by an increase in defect tolerance, as seen by the monotonic reduction in extracted carrier lifespan and diffusion length with Br inclusion. Based on these composition-dependent thermal and defect sensitivity asymmetries, we can derive quantitative design principles for customizing mixed halide perovskites, with the possibility that intermediate Br proportions provide the best efficiency/stability compromise. In a broader sense, this model lays the groundwork for understanding temperature-dependent performance trends in mixed halide perovskite photovoltaics, and it directs future experiments toward optimal device designs based on composition.

## Figures and Tables

**Table 1:** SCAPS-1D simulation parameters for TiO_2_ (electron transport layer), PEDOT:PSS (hole transport layer), and MAPb(Br_x_I_1−x_)_3_ absorber layer for all eleven devices (D1–D11).

Parameter	Unit	TiO_2_ (ETL)	PEDOT:PSS (HTL)	MAPb(Br_x_I_1−x_)_3_
Thickness	nm	50	50	500
Energy bandgap, E_g_	eV	3.20	1.60	see Table 2
Electron affinity, χ	eV	4.00	3.55	see Table 2
Relative permittivity, ε_r_	—	9.0	3.0	see Table 2
CB effective DOS, N_C_	cm^−3^	2.0 × 10^18^	2.2 × 10^18^	2.5 × 10^18^
VB effective DOS, N_V_	cm^−3^	1.8 × 10^19^	1.8 × 10^19^	2.5 × 10^18^
Electron mobility, μ_n_	cm^2^ V^−1^ s^−1^	20	1.0 × 10^−4^	see Table 2
Hole mobility, μ_p_	cm^2^ V^−1^ s^−1^	10	1.0 × 10^−4^	see Table 2
Electron thermal velocity, v_th_,_n_	cm s^−1^	1.0 × 10^7^	1.0 × 10^7^	1.0 × 10^7^
Hole thermal velocity, v_th_,_p_	cm s^−1^	1.0 × 10^7^	1.0 × 10^7^	1.0 × 10^7^
Doping concentrations				
Donor concentration, N_D_	cm^−3^	1.0 × 10^18^	0	0
Acceptor concentration, N_A_	cm^−3^	0	1.0 × 10^18^	0
Defect (trap) parameters				
Defect density, N_t_	cm^−3^	1.0 × 10^15^	1.0 × 10^15^	10^11^–10^20^ (swept)
Trap energy level, E_t_	eV	Mid-gap	Mid-gap	Mid-gap
Capture cross-section (e^−^), σ_n_	cm^2^	1.0 × 10^−15^	1.0 × 10^−15^	1.1 × 10^−15^
Capture cross-section (h^+^), σ_p_	cm^2^	1.0 × 10^−15^	1.0 × 10^−15^	1.1 × 10^−15^
Recombination parameters				
Radiative recombination coefficient, B	cm^3^ s^−1^	0	0	2.3 × 10^−9^
Auger coefficient, α	cm^3^ s^−1^	0	0	1.0 × 10^−29^
Absorption model (Tauc law)				
Absorption constant, A	cm^−1^ eV^−1^/^2^	1.0 × 10^4^	1.0 × 10^4^	1.0 × 10^3^
Contact parameters				
Front contact work function (FTO)	eV	4.40	-	-
Back contact work function (Au)	eV	5.10	-	-
Front surface recombination velocity, v_th_	cm s^−1^	1.0 × 10^7^	-	-
Back surface recombination velocity, v_th_	cm s^−1^	1.0 × 10^7^	-	-
References		[65,66,67]	[68,69,70,71]	[49,50,51,52,53,54,55,56,57,58,59,60,61,62,63,64]

**Table 2:** Material properties of the perovskite absorber layer (MAPb(Br_x_I_1−x_)_3_) as a function of composition derived from Equation (16) for devices D1–D11.

Device	x	Absorber	E_g_ (eV)	χ (eV)	ε_r_	μ_n_ = μ_p_ (cm^2^ V^−1^ s^−1^)
D1	0.0	MAPbI_3_	1.570	3.930	25.5	22.0
D2	0.1	MAPb(Br_0.1_I_0.9_)_3_	1.607	3.912	25.0	21.2
D3	0.2	MAPb(Br_0.2_I_0.8_)_3_	1.651	3.894	24.4	20.3
D4	0.3	MAPb(Br_0.3_I_0.7_)_3_	1.703	3.875	23.8	19.5
D5	0.4	MAPb(Br_0.4_I_0.6_)_3_	1.761	3.856	23.1	18.6
D6	0.5	MAPb(Br_0.5_I_0.5_)_3_	1.824	3.836	22.4	17.7
D7	0.6	MAPb(Br_0.6_I_0.4_)_3_	1.892	3.815	21.7	16.7
D8	0.7	MAPb(Br_0.7_I_0.3_)_3_	1.962	3.794	20.9	15.7
D9	0.8	MAPb(Br_0.8_I_0.2_)_3_	2.034	3.773	20.1	14.6
D10	0.9	MAPb(Br_0.9_I_0.1_)_3_	2.106	3.751	19.3	13.5
D11	1.0	MAPbBr_3_	2.240	3.750	17.0	10.0

## Data Availability

The original contributions presented in this study are included in the article. Further inquiries can be directed to the corresponding author.

## Supplementary Materials

The following supporting information can be downloaded at: https://www.mdpi.com/article/10.3390/nano16150965/s1. Table S1. List of all investigated devices with varying Br fraction (x) in the perovskite absorber layer, alongside TiO_2_ and PEDOT:PSS as ETL (electron-transport layer) and HTL (hole-transport layer). The complete mathematical formulations of the semiconductor transport and recombination models employed in this study, along with the full list of investigated device compositions, are provided in the Supplementary Materials (Equations (S1)–(S15) and Table S1). References [36,37,38,39,40,41,42,43,44,45,46,47,48,55,56,94,95] are cited in the Supplementary Materials.

## References

[1] Green M.A., Dunlop E.D., Yoshita M., Kopidakis N., Bothe K., Siefer G., Hao X. (2024). Solar cell efficiency tables (Version 64). Prog. Photovolt..

[2] Green M.A., Dunlop E.D., Yoshita M., Kopidakis N., Bothe K., Siefer G., Hao X., Jiang J.Y. (2025). Solar cell efficiency tables (version 65). Prog. Photovolt. Res. Appl..

[3] Harit A.K., He Z.F., Kuang Y., Merckx T., Aguirre A., Li M., Xiao K., Wang X., Hou Y., Yan B. (2026). Taking perovskite photovoltaics from promise to product. Nat. Rev. Clean. Technol..

[4] Zhu P., Chen C., Dai J., Zhang Y., Mao R., Chen S., Huang J., Zhu J. (2024). Toward the commercialization of perovskite solar modules. Adv. Mater..

[5] Wang M., Wang W., Ma B., Shen W., Liu L., Cao K., Chen S., Huang W. (2021). Lead-free perovskite materials for solar cells. Nano-Micro Lett..

[6] Huang C., Huang Y., Zhao Q., Liu Z., Shen G., Jiang Q., Luo Y., Li X., Yang J. (2026). Eco-friendly lead-free and low-lead perovskite solar cells. Energy Mater. Devices.

[7] Liu G., Yang G., Feng W., Wu W.Q. (2026). Innovative chemical design and regulation strategies for overcoming lead toxicity in perovskite-based optoelectronics: A new perspective. Chem. Sci..

[8] Han J., Tian Y., Jeon I. (2025). Natural and Nature-Inspired Biomaterial Additives for Metal Halide Perovskite Optoelectronics. Adv. Mater..

[9] Ma S., Yuan G., Zhang Y., Yang N., Li Y., Chen Q. (2022). Development of encapsulation strategies towards the commercialization of perovskite solar cells. Energy Environ. Sci..

[10] Yang W., Zhang Y., Xiao C., Yang J., Shi T. (2025). A review of encapsulation methods and geometric improvements of perovskite solar cells and modules for mass production and commercialization. Nano Mater. Sci..

[11] Li J., Geng M., Jiang L., Xu T. (2026). Advances of Self-Healing Polymers Incorporated in Perovskite Solar Cells for High Durability. Nano-Micro Lett..

[12] Yu Y., Zhang F., Yu H. (2020). Self-healing perovskite solar cells. Sol. Energy.

[13] Xu W., Zheng L., Zhang X., Cao Y., Meng T., Wu D., Liu L., Hu W., Gong X. (2018). Efficient perovskite solar cells fabricated by Co partially substituted hybrid perovskite. Adv. Energy Mater..

[14] Zhou Y., Wang F., Fang H.H., Loi M.A., Xie F.Y., Zhao N., Wong C.P. (2016). Distribution of bromine in mixed iodide–bromide organolead perovskites and its impact on photovoltaic performance. J. Mater. Chem. A.

[15] Yang M., Wang H., Cai W., Zang Z. (2023). Mixed-halide inorganic perovskite solar cells: Opportunities and challenges. Adv. Opt. Mater..

[16] Jeon N.J., Noh J.H., Yang W.S., Kim Y.C., Ryu S., Seo J., Seok S.I. (2015). Compositional engineering of perovskite materials for high-performance solar cells. Nature.

[17] Cordero F., Craciun F., Paoletti A.M., Zanotti G. (2021). Structural Transitions and Stability of FAPbI_3_ and MAPbI_3_: The Role of Interstitial Water. Nanomaterials.

[18] Pérez-Gutiérrez E., Percino M.J., Santos P., Cerón M., Ceballos P., Montoya D.M., Barbosa-García O., Thamotharan S. (2020). Compositional study of mixed halide perovskite films CH_3_NH_3_Pb(I_1-x_Br_x_)_3_ and CH_3_NH_3_Pb(I_1-x_Cl_x_)_3_ prepared by close space sublimation. Mater. Today Commun..

[19] García-Rodríguez R., Ferdani D., Pering S., Baker P.J., Cameron P.J. (2019). Influence of bromide content on iodide migration in inverted MAPb (I_1−x_Br_x_)_3_ perovskite solar cells. J. Mater. Chem. A.

[20] McGovern L., Grimaldi G., Futscher M.H., Hutter E.M., Muscarella L.A., Schmidt M.C., Ehrler B. (2021). Reduced barrier for ion migration in mixed-halide perovskites. ACS Appl. Energy Mater..

[21] Muscarella L.A., Hutter E.M., Wittmann F., Woo Y.W., Jung Y.K., McGovern L., Versluis J., Walsh A., Bakker H.J., Ehrler B. (2020). Lattice compression increases the activation barrier for phase segregation in mixed-halide perovskites. ACS Energy Lett..

[22] Xu F., Zhang M., Li Z., Yang X., Zhu R. (2023). Challenges and perspectives toward future wide-bandgap mixed-halide perovskite photovoltaics. Adv. Energy Mater..

[23] Akin S., Arora N., Zakeeruddin S.M., Grätzel M., Friend R.H., Dar M.I. (2020). New strategies for defect passivation in high-efficiency perovskite solar cells. Adv. Energy Mater..

[24] Zhao Z., Wu J., Zheng Y.Z., Li N., Li X., Ye Z., Lu S., Tao X., Chen C. (2019). Stable hybrid perovskite MAPb (I_1−x_Br_x_)_3_ for photocatalytic hydrogen evolution. Appl. Catal. B Environ..

[25] Li C., Li H., Zhu Z., Cui N., Tan Z.A., Yang R. (2021). Perovskite passivation strategies for efficient and stable solar cells. Sol. RRL.

[26] Minemoto T., Murata M. (2015). Theoretical analysis on effect of band offsets in perovskite solar cells. Sol. Energy Mater. Sol. Cells.

[27] Saha N., Brunetti G., Carlo A.D., Ciminelli C. (2025). Efficiency boost of perovskite solar cell in homojunction configuration through tailored band alignment and p–n doping profile. J. Phys. Energy.

[28] Bommidi R.G., Reddy C.K. (2026). Transition to Pb-reduced MAPbI_3_ perovskite solar cells via bandgap grading using MASnI_3_ front and MAGeI_3_ back absorbers. Solid State Commun..

[29] Paz Totolhua E., Carrillo López J., Hernández de la Luz J.Á.D., Monfil Leyva K., Flores-Méndez J., Piñón Reyes A.C., Hernández Simón Z.J., Luna López J.A. (2025). Enhanced efficiency of mixed-halide perovskite solar cells through optimization of the layer thicknesses, defect density, and metal contact work function. Materials.

[30] Saidani O., Goumri-Said S., Yousfi A., Sahoo G.S., Kanoun M.B. (2025). Probing high-efficiency Cs_0.05_ (FA_0.77_MA_0.23_) 0.95 Pb (I_0.77_Br_0.23_)_3_-based perovskite solar cells through first principles computations and SCAPS-1D simulation. RSC Adv..

[31] Ozurumba A.C., Ogueke N.V., Madu C.A., Danladi E., Mbachu C.P., Yusuf A.S., Gyuk P.M., Hossain I. (2024). SCAPS-1D simulated organometallic halide perovskites: A comparison of performance under Sub-Saharan temperature condition. Heliyon.

[32] Burgelman M., Decock K., Khelifi S., Abass A. (2013). Advanced electrical simulation of thin film solar cells. Thin Solid Film..

[33] Burgelman M., Verschraegen J., Degrave S., Nollet P. (2004). Modeling thin-film PV devices. Prog. Photovolt. Res. Appl..

[34] Verschraegen J., Burgelman M. (2007). Numerical modeling of intra-band tunneling for heterojunction solar cells in scaps. Thin Solid Film..

[35] Moiz S.A. (2021). Optimization of hole and electron transport layer for highly efficient lead-free Cs_2_TiBr_6_-based perovskite solar cells. Photonics.

[36] Kingston R.H., Neustadter S.F. (1955). Calculation of the space charge, electric field, and free carrier concentration at the surface of a semiconductor. J. Appl. Phys..

[37] Gray J.L. (2011). The physics of the solar cell. Handbook of Photovoltaic Science and Engineering.

[38] Fonash S.J. (2012). Solar Cell Device Physics.

[39] Chin V.J., Salam Z., Ishaque K. (2015). Cell modelling and model parameters estimation techniques for photovoltaic simulator application: A review. Appl. Energy.

[40] Burgelman M., Decock K., Niemegeers A., Verschraegen J., Degrave S. (2016). SCAPS Manual.

[41] Compaan K., Haven Y. (1956). Correlation factors for diffusion in solids. Trans. Faraday Soc..

[42] Roichman Y., Tessler N. (2002). Generalized Einstein relation for disordered semiconductors—Implications for device performance. Appl. Phys. Lett..

[43] Zeman M., Krc J. (2008). Optical and electrical modeling of thin-film silicon solar cells. J. Mater. Res..

[44] Alkauskas A., Dreyer C.E., Lyons J.L., Van de Walle C.G. (2016). Role of excited states in Shockley-Read-Hall recombination in wide-band-gap semiconductors. Phys. Rev. B.

[45] Sah C.T., Noyce R.N., Shockley W. (1957). Carrier generation and recombination in pn junctions and pn junction characteristics. Proc. IRE.

[46] Ahmed M.M., Karimov K.S., Moiz S.A. (2008). Photoelectric behavior of n-GaAs/orange dye, vinyl-ethynyl-trimethyl-piperidole/conductive glass sensor. Thin Solid Films.

[47] de Quilettes D.W., Vorpahl S.M., Stranks S.D., Nagaoka H., Eperon G.E., Ziffer M.E., Snaith H.J., Ginger D.S. (2015). Impact of microstructure on local carrier lifetime in perovskite solar cells. Science.

[48] Dequilettes D.W., Frohna K., Emin D., Kirchartz T., Bulovic V., Ginger D.S., Stranks S.D. (2019). Charge-carrier recombination in halide perovskites: Focus review. Chem. Rev..

[49] Schulz P., Edri E., Kirmayer S., Hodes G., Cahen D., Kahn A. (2014). Interface energetics in organo-metal halide perovskite-based photovoltaic cells. Energy Environ. Sci..

[50] Miller E.M., Zhao Y., Mercado C.C., Saha S.K., Luther J.M., Zhu K., Stevanović V., Perkins C.L., van de Lagemaat J. (2014). Substrate-controlled band positions in CH_3_NH_3_PbI_3_ perovskite films. Phys. Chem. Chem. Phys..

[51] Brivio F., Butler K.T., Walsh A., Van Schilfgaarde M. (2014). Relativistic quasiparticle self-consistent electronic structure of hybrid halide perovskite photovoltaic absorbers. Phys. Rev. B.

[52] Butler K.T., McKechnie S., Azarhoosh P., Van Schilfgaarde M., Scanlon D.O., Walsh A. (2016). Quasi-particle electronic band structure and alignment of the V-VI-VII semiconductors SbSI, SbSBr, and SbSeI for solar cells. Appl. Phys. Lett..

[53] Frost J.M., Butler K.T., Brivio F., Hendon C.H., Van Schilfgaarde M., Walsh A. (2014). Atomistic origins of high-performance in hybrid halide perovskite solar cells. Nano Lett..

[54] Noh J.H., Im S.H., Heo J.H., Mandal T.N., Seok S.I. (2013). Chemical management for colorful, efficient, and stable inorganic–organic hybrid nanostructured solar cells. Nano Lett..

[55] Adjokatse S., Fang H.H., Loi M.A. (2017). Broadly tunable metal halide perovskites for solid-state light-emission applications. Mater. Today.

[56] Mancini A., Quadrelli P., Milanese C., Patrini M., Guizzetti G., Malavasi L. (2015). CH_3_NH_3_Sn_x_Pb_1–x_Br_3_ Hybrid Perovskite Solid Solution: Synthesis, Structure, and Optical Properties. Inorg. Chem..

[57] Mahapatra A., Prochowicz D., Kruszyńska J., Satapathi S., Akin S., Kumari H., Kumar P., Fazel Z., Tavakoli M.M., Yadav P. (2021). Effect of bromine doping on the charge transfer, ion migration and stability of the single crystalline MAPb (Br_x_I_1−x_)_3_ photodetector. J. Mater. Chem. C.

[58] Bawazir H.S., Qaid S.M., Ghaithan H.M., AlHarbi K.K., Bin Ajaj A.F., Aldwayyan A.S. (2022). Phase state influence on photoluminescence of MAPb (BrxI_1−x_)_3_ perovskites towards optimized photonics applications. Photonics.

[59] Zhang Y., Liu Y., Li Y., Yang Z., Liu S.F. (2016). Perovskite CH_3_NH_3_Pb (Br_x_I_1−x_)_3_ single crystals with controlled composition for fine-tuned bandgap towards optimized optoelectronic applications. J. Mater. Chem. C.

[60] Chen X., Han D., Su Y., Zeng Q., Liu L., Shen D. (2018). Structural and electronic properties of inorganic mixed halide perovskites. Phys. Status Solidi (RRL) Rapid Res. Lett..

[61] Ghaithan H.M., Qaid S.M., Alahmed Z.A., Hezam M., Lyras A., Amer M., Aldwayyan A.S. (2021). Anion Substitution Effects on the Structural, Electronic, and Optical Properties of Inorganic CsPb (I_1−x_Br_x_)_3_ and CsPb (Br_1−x_Cl_x_)_3_ Perovskites: Theoretical and Experimental Approaches. J. Phys. Chem. C.

[62] Ghaithan H.M., Alahmed Z.A., Qaid S.M., Aldwayyan A.S. (2020). Structural, electronic, and optical properties of CsPb (Br_1−x_Cl_x_)_3_ perovskite: First-principles study with PBE–GGA and mBJ–GGA methods. Materials.

[63] Lei S., An B., Peng G., Chen X., Jia H., Jin Z. (2026). Physics-Aware Hybrid Equivariant Graph Neural Networks for Robust Design of Inorganic Perovskite Photodetector. Adv. Opt. Mater..

[64] Suchan K., Just J., Becker P., Rehermann C., Merdasa A., Mainz R., Scheblykin I.G., Unger E.L. (2021). Broad distribution of local I/Br ratio in illuminated mixed halide perovskite films revealed by correlative X-ray diffraction and photoluminescence. arXiv.

[65] Usman A., Bovornratanaraks T. (2024). Modeling and optimization of modified TiO_2_ with aluminum and magnesium as ETL in MAPbI_3_ perovskite solar cells: SCAPS 1D frameworks. ACS Omega.

[66] Moiz S.A., Masud M.I. (2025). Enhancing CaV_0.5_Fe_0.5_O_3_-Based Lead-Free Perovskite Solar Cell Efficiency by over 23% via Transport Layer Engineering. Nanomaterials.

[67] Zaleska A. (2008). Doped-TiO_2_: A review. Recent Pat. Eng..

[68] Alipour H., Ghadimi A. (2021). Optimization of lead-free perovskite solar cells in normal-structure with WO3 and water-free PEDOT: PSS composite for hole transport layer by SCAPS-1D simulation. Opt. Mater..

[69] Mandadapu U., Vedanayakam S.V., Thyagarajan K. (2017). Simulation and analysis of lead based perovskite solar cell using SCAPS-1D. Indian J. Sci. Technol..

[70] Moiz S.A., Alshaikh M.S., Alahmadi A.N. (2023). Simulation design of novel non-fluorine polymers as electron transport layer for lead-free Perovskite solar cells. Polymers.

[71] Moiz S.A., Alahmadi A.N.M., Aljohani A.J. (2021). Design of a novel lead-free perovskite solar cell for 17.83% efficiency. IEEE Access.

[72] Lee J., Park J., Jeon B.C., Lim S., Back J.Y., Kim J., Moon T. (2024). Employing PEDOT: PSS as a hole transport material in regular rudorffite AgBiI4 solar cells for indoor photovoltaics. ACS Sustain. Chem. Eng..

[73] Yi H., Wang D., Duan L., Haque F., Xu C., Zhang Y., Conibeer G., Uddin A. (2019). Solution-processed WO3 and water-free PEDOT: PSS composite for hole transport layer in conventional perovskite solar cell. Electrochim. Acta.

[74] Makha M., Fernandes S.L., Jenatsch S., Offermans T., Schleuniger J., Tisserant J.N., Véron A.C., Hany R. (2016). A transparent, solvent-free laminated top electrode for perovskite solar cells. Sci. Technol. Adv. Mater..

[75] Koushik D., Verhees W.J., Zhang D., Kuang Y., Veenstra S., Creatore M., Schropp R.E. (2017). Atomic layer deposition enabled perovskite/PEDOT solar cells in a regular n–i–p architectural design. Adv. Mater. Interfaces.

[76] Liu J., Pathak S., Stergiopoulos T., Leijtens T., Wojciechowski K., Schumann S., Kausch-Busies N., Snaith H.J. (2015). Employing PEDOT as the p-type charge collection layer in regular organic–inorganic perovskite solar cells. J. Phys. Chem. Lett..

[77] Shi C., Li J., Xiao S., Wang Z., Xiang W., Wu R., Liu Y., Zhou Y., Ke W., Fang G. (2024). Alcohol-dispersed polymer complex as an effective and durable interface modifier for nip perovskite solar cells. J. Energy Chem..

[78] Jiang Y., Luo B., Jiang F., Jiang F., Fuentes-Hernandez C., Liu T., Mao L., Xiong S., Li Z., Wang T. (2016). Efficient colorful perovskite solar cells using a top polymer electrode simultaneously as spectrally selective antireflection coating. Nano Lett..

[79] Kegelmann L., Tockhorn P., Wolff C.M., Márquez J.A., Caicedo-Dávila S., Korte L., Unold T., Lövenich W., Neher D., Rech B. (2019). Mixtures of dopant-free spiro-OMeTAD and water-free PEDOT as a passivating hole contact in perovskite solar cells. ACS Appl. Mater. Interfaces.

[80] Forgacs D., Perez-del-Rey D., Avila J., Momblona C., Gil-Escrig L., Dänekamp B., Sessolo M., Bolink H.J. (2017). Efficient wide band gap double cation–double halide perovskite solar cells. J. Mater. Chem. A.

[81] Moiz S.A., Alahmadi A.N., Alshaikh M.S. (2025). Design Optimization of Cesium Contents for Mixed Cation MA_1−x_Cs_x_PbI_3_-Based Efficient Perovskite Solar Cell. Nanomaterials.

[82] Sarkar J., Talukdar A., Debnath P., Chatterjee S. (2023). Study of bromine substitution on band gap broadening with consequent blue shift in optical properties and efficiency optimization of lead-free CsGeI_X_Br_3−X_ based perovskite solar cells. J. Comput. Electron..

[83] Schilinsky P., Waldauf C., Brabec C.J. (2002). Recombination and loss analysis in polythiophene based bulk heterojunction photodetectors. Appl. Phys. Lett..

[84] Landi G., Neitzert H.C., Barone C., Mauro C., Lang F., Albrecht S., Rech B., Pagano S. (2017). Correlation between electronic defect states distribution and device performance of perovskite solar cells. Adv. Sci..

[85] Moiz S.A., Khan I.A., Younis W.A., Masud M.I., Ismail Y., Khawaja Y.M. (2020). Solvent induced charge transport mechanism for conducting polymer at higher temperature. Mater. Res. Express.

[86] Meggiolaro D., Motti S.G., Mosconi E., Barker A.J., Ball J., Perini C.A.R., Deschler F., Petrozza A., De Angelis F. (2018). Iodine chemistry determines the defect tolerance of lead-halide perovskites. Energy Environ. Sci..

[87] Taheri S., Minbashi M., Hajjiah A. (2021). Effect of defects on high efficient perovskite solar cells. Opt. Mater..

[88] Caprioglio P., Stolterfoht M., Wolff C.M., Unold T., Rech B., Albrecht S., Neher D. (2019). On the relation between the open-circuit voltage and quasi-fermi level splitting in efficient perovskite solar cells. Adv. Energy Mater..

[89] Stolterfoht M., Wolff C.M., Amir Y., Paulke A., Perdigón-Toro L., Caprioglio P., Neher D. (2017). Approaching the fill factor Shockley–Queisser limit in stable, dopant-free triple cation perovskite solar cells. Energy Environ. Sci..

[90] Kostylyov V.P., Sachenko A.V., Evstigneev M., Sokolovskyi I.O., Shkrebtii A.I. (2025). Characterization and optimization of high-efficiency crystalline silicon solar cells: Impact of recombination in the space charge region and trap-assisted Auger exciton recombination. J. Appl. Phys..

[91] Zhang M., Yang M., Yang Z., Yang B., Lei L., Chen H., Duan J., He B. (2026). Phase Segregation Mechanism and Suppression in Wide-Bandgap Perovskites toward Efficient and Stable Perovskite/Silicon Tandem Solar Cells. Sustain. Chem. Energy Mater..

[92] Brakkee R., Williams R.M. (2020). Minimizing defect states in lead halide perovskite solar cell materials. Appl. Sci..

[93] Fossum J.G., Lee D.S. (1982). A physical model for the dependence of carrier lifetimes on doping density in nondegenerate silicon. Solid-State Electron..

[94] Cuevas A., Macdonald D. (2004). Measuring and interpreting the lifetime of silicon wafers. Sol. Energy.

[95] Adhikari R.D., Patel M.J., Baishya H., Yadav D., Kalita M., Alam M., Iyer P.K. (2025). Decoding recombination dynamics in perovskite solar cells: An in-depth critical review. Chem. Soc. Rev..

[96] Moiz S.A., Alahmadi A.N.M., Aljohani A.J. (2020). Design of silicon nanowire array for PEDOT: PSS-silicon nanowire-based hybrid solar cell. Energies.

[97] Yu Y., Liu X., Zhang S., Chen J. (2024). Photoinduced phase segregation in wide-bandgap mixed-halide perovskite solar cells. Energy Mater. Devices.

[98] Schwenzer J.A., Rakocevic L., Gehlhaar R., Abzieher T., Gharibzadeh S., Moghadamzadeh S., Quintilla A., Richards B.S., Lemmer U., Paetzold U.W. (2018). Temperature variation-induced performance decline of perovskite solar cells. ACS Appl. Mater. Interfaces.

[99] Rana A., Sharma P., Kumar A., Pareek S., Waheed S., Singh R.K., Karak S. (2021). Understanding the correlation between temperature dependent performance and trap distribution for nickel oxide based inverted perovskite solar cells. IEEE Trans. Electron. Devices.

[100] Moiz S.A., Karimov K.S., Gohar N.D. (2004). Orange dye thin film resistive hygrometers. Eurasian Chem.-Technol. J..

