# Supplementary material for: Design of MAPb(BrxI1−x)3-Based Solar Cells: Compositional Optimization for Thermally Stable and Defect-Tolerant Devices
Source: Nanomaterials (Basel). 2026 Aug 6;16(15):965. doi: 10.3390/nano16150965 (PMC13467878; doi:10.3390/nano16150965)
Supplement: Supplementary file 1 [file nanomaterials-16-00965-s001.zip › nanomaterials-4447112-supplementary.pdf]

Supplementary Materials

Design of $MAPb(Br_xI_{1-x})_3$-Based Solar Cells: Compositional Optimization for Thermally Stable and Defect-Tolerant Devices

Syed Abdul Moiz [1,*], Muhammad I. Masud [2] and Muhammad Kashif [3]

[1] Device Simulation Laboratory, Department of Electrical Engineering, College of Engineering and Architecture, Umm Al-Qura University, Makkah 21955, Saudi Arabia
[2] Department of Electrical Engineering, College of Engineering, University of Business and Technology, Jeddah 21361, Saudi Arabia; m.masud@ubt.edu.sa
[3] School of Electrical and Information Engineering, 92 Weijin Road, Nankai District, Tianjin University, Tianjin 300072, China; mkashif@tju.edu.cn
* Correspondence: sasyed@uqu.edu.sa

S1. Complete List of Investigated Devices

Table S1. List of all investigated devices with varying Br fraction (x) in the perovskite absorber layer, alongside TiO_2 and PEDOT:PSS as ETL (electron-transport layer) and HTL (hole-transport layer).

Device	x	Perovskite Composition	ETL	HTL
D1	0.0	$MAPbI_3$	TiO_2	PEDOT:PSS
D2	0.1	$MAPb(Br_{0.1}I_{0.9})_3$	TiO_2	PEDOT:PSS
D3	0.2	$MAPb(Br_{0.2}I_{0.8})_3$	TiO_2	PEDOT:PSS
D4	0.3	$MAPb(Br_{0.3}I_{0.7})_3$	TiO_2	PEDOT:PSS
D5	0.4	$MAPb(Br_{0.4}I_{0.6})_3$	TiO_2	PEDOT:PSS
D6	0.5	$MAPb(Br_{0.5}I_{0.5})_3$	TiO_2	PEDOT:PSS
D7	0.6	$MAPb(Br_{0.6}I_{0.4})_3$	TiO_2	PEDOT:PSS
D8	0.7	$MAPb(Br_{0.7}I_{0.3})_3$	TiO_2	PEDOT:PSS
D9	0.8	$MAPb(Br_{0.8}I_{0.2})_3$	TiO_2	PEDOT:PSS
D10	0.9	$MAPb(Br_{0.9}I_{0.1})_3$	TiO_2	PEDOT:PSS
D11	1.0	$MAPbBr_3$	TiO_2	PEDOT:PSS

S2. Mathematical Models Employed for Simulation

SCAPS-1D self-consistently integrates a connected framework of fundamental semiconductor models to simulate the steady-state performance of solar cell devices. The detailed mathematical formulations employed in this study are provided below.

S2.1 Poisson Equation

The primary governing equation is the Poisson equation, which connects the electrostatic potential ψ to the spatial charge density distributions within the device [29–32]:

$$\frac{d^2\psi}{d^2x} = -\frac{q}{\varepsilon_0\varepsilon_r}(p - n + N_D - N_A + \rho_t) \quad \text{(S1)}$$

where:

- q = electrical charge constant (1.602×10^{-19} C)
- ε_0 = permittivity of free space
- ε_r = relative dielectric permittivity of the material
- n and p = concentrations of free electrons and holes
- N_D and N_A = concentrations of ionized donors and acceptors
- ρ_t = charge density attributed to trapped carriers

S2.2 Continuity Equations

The continuity equations for electrons and holes ensure carrier conservation by balancing generation, recombination, and drift-diffusion transport [29–32]:

$$\frac{1}{q}\frac{dJ_n}{dx} = G - R \quad \text{(S2)}$$

$$\frac{1}{q}\frac{dJ_p}{dx} = G - R \quad \text{(S3)}$$

where:

- J_n and J_p = electron and hole current densities
- G = free carrier generation rate of electron-hole pairs
- R = net recombination rate

S2.3 Drift-Diffusion Transport Equations

The current densities of electrons and holes are defined using the drift-diffusion charge transport equations [29–32]:

$$J_n = q\mu_n n\frac{d\psi}{dx} + qD_n\frac{dn}{dx} \quad \text{(S4)}$$

$$J_P = q\mu_P p \frac{d\psi}{dx} - qD_p \frac{dp}{dx} \quad \text{(S5)}$$

where:

- μ_n and μ_p = electron and hole mobilities
- D_n and D_p = diffusion coefficients for electrons and holes

---

S2.4 Einstein Relation

The diffusion coefficients are connected to mobility by the Einstein relation [34–36]:

$$D_n = \frac{k_B T}{q}\mu_n \quad \text{(S6)}$$

$$D_p = \frac{k_B T}{q}\mu_p \quad \text{(S7)}$$

where:

- T = ambient temperature
- k_B = Boltzmann constant

S2.5 Shockley-Read-Hall (SRH) Recombination

The primary recombination process in $MAPb(Br_xI_{1-x})_3$ perovskite absorbers is Shockley-Read-Hall (SRH) trap-assisted recombination [37–39]:

$$R_{SRH} = \frac{np - n_i^2}{\tau_p(n + n_i) + \tau_n(p + p_1)} \quad \text{(S8)}$$

where:

- n_i = intrinsic carrier concentration
- τ_n and τ_p = electron and hole carrier lifetimes
- n_1 and p_1 = electron and hole concentrations when Fermi level aligns with trap energy level

The carrier lifetimes are associated with trap density N_t and capture cross-sections σ_n and σ_p [40]:

$$\tau_n = \frac{1}{\sigma_n v_{th} N_t} \quad \text{(S9)}$$

$$\tau_p = \frac{1}{\sigma_p v_{th} N_t} \quad \text{(S10)}$$

where v_{th} denotes the thermal velocity of charge carriers.

The variables n_1 and p_1 are defined as:

$$n_1 = n_i e^{\left(\frac{E_t - E_i}{k_B T}\right)} \quad \text{(S11)}$$

$p_1 = n_i e^{\left(\frac{E_i - E_t}{k_B T}\right)}$ (S12)

where E_i represents the intrinsic Fermi level of energy.

S2.6 Radiative Recombination

Radiative band-to-band recombination is included in SCAPS-1D as [41]:

$R_{rad} = B(np - n_i^2)$ (S13)

where B indicates the radiative recombination (absorption) coefficient.

S2.7 Total Recombination Rate

The overall recombination rate R can be defined as:

$R = R_{SRH} + R_{rad}$ (S14)

S2.8 Generation Rate

The generation rate of free carriers $G(x)$, as a function of thickness x, is determined from the Beer-Lambert law using the incident AM1.5G solar spectrum [30]:

$G(\lambda) = \int_{\lambda} \alpha(\lambda)\phi(\lambda)\, e^{(-\alpha(\lambda)x)} d\lambda$ (S15)

where:

- $\alpha(\lambda)$ = wavelength-dependent absorption coefficient
- $\Phi(\lambda)$ = incoming photon flux density at wavelength λ

References for Supplementary Materials

29. Kingston, R. H., & Neustadter, S. F. (1955). Calculation of the space charge, electric field, and free carrier concentration at the surface of a semiconductor. *Journal of Applied Physics*, *26*(6), 718-720.

30. Gray, J.L. The physics of the solar cell. In *Handbook of Photovoltaic Science and Engineering*; Wiley: Hoboken, NJ, USA, 2011; Volume 2, pp. 82–128.

31. Fonash, S.J. *Solar Cell Device Physics*; Elsevier: Amsterdam, The Netherlands, 2012.

32. Chin, V.J.; Salam, Z.; Ishaque, K. Cell modelling and model parameters estimation techniques for photovoltaic simulator application: A review. *Appl. Energy* 2015, *154*, 500–519.

33. Burgelman, M.; Decock, K.; Niemegeers, A.; Verschraegen, J.; Degrave, S. *SCAPS Manual*; University of Ghent: Ghent, Belgium, 2016.

34. Compaan, K., & Haven, Y. (1956). Correlation factors for diffusion in solids. *Transactions of the Faraday Society*, *52*, 786-801.

35. Roichman, Y., & Tessler, N. (2002). Generalized Einstein relation for disordered semiconductors—implications for device performance. *Applied Physics Letters*, *80*(11), 1948-1950.

36. Zeman, M.; Krc, J. Optical and electrical modeling of thin-film silicon solar cells. *J. Mater. Res.* 2008, *23*, 889–898.

37. Alkauskas, A., Dreyer, C. E., Lyons, J. L., & Van de Walle, C. G. (2016). Role of excited states in Shockley-Read-Hall recombination in wide-band-gap semiconductors. *Physical Review B*, *93*(20), 201304.

38. Sah, C. T., Noyce, R. N., & Shockley, W. (1957). Carrier generation and recombination in pn junctions and pn junction characteristics. *Proceedings of the IRE*, *45*(9), 1228-1243.

39. Ahmed, M. M., Karimov, K. S., & Moiz, S. A. (2008). Photoelectric behavior of n-GaAs/orange dye, vinyl-ethynyl-trimethyl-piperidole/conductive glass sensor. *Thin Solid Films*, *516*(21), 7822-7827.

40. de Quilettes, D. W., Vorpahl, S. M., Stranks, S. D., Nagaoka, H., Eperon, G. E., Ziffer, M. E., ... & Ginger, D. S. (2015). Impact of microstructure on local carrier lifetime in perovskite solar cells. *Science*, *348*(6235), 683-686.

41. Dequilettes, D. W., Frohna, K., Emin, D., Kirchartz, T., Bulovic, V., Ginger, D. S., & Stranks, S. D. (2019). Charge-carrier recombination in halide perovskites: Focus review. *Chemical reviews*, *119*(20), 11007-11019.

42. Adjokatse, S., Fang, H. H., & Loi, M. A. (2017). Broadly tunable metal halide perovskites for solid-state light-emission applications. *Materials Today*, *20*(8), 413-424.

43. Mancini, A., Quadrelli, P., Milanese, C., Patrini, M., Guizzetti, G., & Malavasi, L. (2015). CH3NH3SnxPb1−xBr3 Hybrid Perovskite Solid Solution: Synthesis, Structure, and Optical Properties. *Inorganic Chemistry*, *54*(18), 8893-8895.

44. Cuevas, A., & Macdonald, D. (2004). Measuring and interpreting the lifetime of silicon wafers. *Solar Energy*, *76*(1-3), 255-262.

45. Adhikari, R. D., Patel, M. J., Baishya, H., Yadav, D., Kalita, M., Alam, M., & Iyer, P. K. (2025). Decoding recombination dynamics in perovskite solar cells: an in-depth critical review. *Chemical Society Reviews*, *54*(8), 3962-4034.
